# Quantitative analysis of exosomes in the aqueous humor of Korean patients with pseudoexfoliation glaucoma

**DOI:** 10.1038/s41598-022-17063-9

**Published:** 2022-07-27

**Authors:** Hyo Jung An, Hyun-kyung Cho, Dae Hyun Song, Changwon Kee

**Affiliations:** 1 Department of Pathology, Gyeongsang National University Changwon Hospital, Gyeongsang National University, School of Medicine, Changwon, Republic of Korea; 2 Institute of Health Sciences, School of Medicine, Gyeongsang National University, Jinju, Republic of Korea; 3 Department of Ophthalmology, Gyeongsang National University Changwon Hospital, Gyeongsang National University, School of Medicine, 11 Samjeongja-ro, Seongsan-gu, Changwon, Gyeongsangnam-do 51472 Republic of Korea; 4 Department of Ophthalmology, Samsung Medical Center, Sungkyunkwan University School of Medicine, Seoul, Republic of Korea

**Keywords:** Nanoparticles, Optic nerve diseases

## Abstract

We aimed to quantitatively analyze the exosome and its cargo in individual aqueous humor (AH) samples from pseudoexfoliation (PEX) glaucoma patients compared to controls using a novel detection platform. We investigated the size distribution and measured the quantitative exosome particle counts in each AH sample. AH (80–120 µL) was obtained during cataract surgery or glaucoma filtering surgery from 12 Korean subjects (six with PEX glaucoma and six age-matched controls). The mean size of the exosomes was 58.9 ± 18.5 nm measured by a tangential flow filtration system using single-particle interferometric reflectance imaging sensor. Exosome particle count in each CD 63, CD 81, and CD9 spot was significantly greater in PEX glaucoma than in controls in total, CD 63, CD9, syntenin, and scattering(all *p* < 0.003). The CD63 spot showed a particle count of 8319.1 ± 797.7 in PEX glaucoma patients and 4786.8 ± 1302.1 in controls (*p* = 1.88E−11). Individual fluorescent capture spot images also revealed denser exosome particles in PEX patients than in controls. Syntenin, indicating exosomal origin, was detected in all AH samples. Exosomes differentially detected in AH suggest the possible role of exosomes in the pathogenesis of PEX glaucoma.

## Introduction

Glaucoma is the second most common cause of visual deficit in the world, which ultimately can bring about blindness^1,2^. The recent global population with primary open-angle glaucoma (POAG) is approximated to be 68.56 million^1^. Asians comprise half (53.81%) of the POAG patients^1^. Glaucoma, as a neurodegenerative disease, has pathological characteristics of retinal ganglion cell (RGC) death^3^. Pseudoexfoliation (PEX) syndrome is a disorder related to age and associated with extracellular matrix. It is the most common distinguishable cause of secondary glaucoma, called PEX glaucoma^4^. It features progressive accumulation of white fibrillar materials in various extra- and intra-ocular tissues^4^. PEX glaucoma is usually more aggressive and the response to hypotensive medications is less than POAG^4^. It also exibits higher intraocular pressure (IOP). The pathophysiology of PEX syndrome has not yet been uncovered, even though it is speculated to have a genetic basis^5–7^.

Exosomes are small vesicles which have a diameter of approximately 30–150 nm and are enclosed by a double-layered lipid membrane^8^. They carry proteins, mRNA, or miRNA and transport them to adjacent or distant cells^9^. Exosomes are secreted by several types of cells and discovered in numerous body fluids, including blood plasma, urine, and aqueous humor (AH)^10–12^. The main function of exosomes involves intercellular communication by transporting exosomal RNA and protein between cells. Therefore, exosomes have emerged as important biomarkers in a number of human diseases^11,13,14^.

AH has the main role of providing nutrition, removing excretory products from metabolism, transporting neurotransmitters, and contributing to the regulation of homeostasis in ocular tissues^15^. Latest studies proposed that there are potential modulators in AH, for example, growth factors and hormones, which may be involved in cellular communication between ocular tissues^16–18^. These regulators are related with the pathogenesis of numerous eye diseases^14,19,20^.

Exosomes were detected in AH in previous studies^12,19,21^. Among them, Dismuke et al. collected exosomes from human AH during surgery on cataract patients and isolated them using ultracentrifugation^12^. They extracted RNA from pooled human AH and discovered and hypothesized that miRNAs within exosomes may assist in communication between AH inflow and outflow tissue^12^. However, they did not include patients with specific ocular diseases such as glaucoma. They only included cataract patients undergoing cataract surgery or those with diabetes or myopia. Thus, the role of exosomes in a specific ocular disease like glaucoma has not been investigated. Furthermore, exosomes from the AH of the specific glaucoma type PEX glaucoma have not been investigated before, especially in a single ethnic group of Koreans.

Previous studies did not use a novel detection platform based on a single-particle interferometric reflectance imaging sensor and a tangential flow filtration system-based method for high-yield separation and the analysis of exosomal bioactivity. Using this method, small amounts of individual AH samples could be analyzed without pooling all samples. Moreover, this novel detection platform could provide the quantitative analysis of particle counts, in addition to analyzing the size and cargo.

In the present study, we investigated the size distribution and quantified exosome particles in AH from PEX glaucoma patients compared to controls using a novel detection platform in Koreans without pooling the samples. We intended to see differentially detected exosomes, which might suggest a role of exosomes in the pathogenesis of PEX glaucoma or might provide some clues to the pathogenesis of PEX glaucoma.

## Results

### Baseline characteristics and demographics of the subjects

Six PEX patients and six age-matched control subjects were included in the final analysis. The demographics of the included subjects are presented in Table 1. The mean age of the PEX glaucoma subjects was 73.3 ± 9.9 years (*n* = 6) and 69.3 ± 38.1 years for the control subjects (*n* = 6). The mean baseline IOP was 30.7 ± 4.3 mmHg in the PEX glaucoma subjects and 13.2 ± 1.9 mmHg in the control subjects. The baseline mean deviation (MD) was -24.6 ± 4.7 dB in the PEX glaucoma group. The PEX glaucoma patients were using multiple topical anti-glaucoma medications (all three medications) before glaucoma filtering surgery. No control subjects were using topical anti-glaucoma medications. The included subjects had no ocular comorbidities other than simple cataracts.

**Table 1:** Baseline characteristics and demographics of subjects.

Subject number	Disease status	Age, y	Sex	Eye laterality	Mean IOP, mmHg	Topical medication	Type of surgery
1	Control	65	Female	Right	12	None	Phaco + PCL
2	Control	75	Male	Left	15	None	Phaco + PCL
3	Control	68	Female	Right	14	None	Phaco + PCL
4	Control	75	Male	Right	10	None	Phaco + PCL
5	Control	62	Female	Left	13	None	Phaco + PCL
6	Control	47	Male	Left	15	None	Phaco + PCL
7	PEX G	67	Male	Left	26	Timolol/bimatoprost, brinzolamide, brimonidine	Ahmed valve
8	PEX G	58	Male	Left	25	Dorzolamide/timolol, brimonidine, bimatoprost	Ahmed valve
9	PEX G	81	Female	Left	33	Dorzolamide/timolol, brimonidine, bimatoprost	Ahmed valve
10	PEX G	83	Male	Left	31	Brinzolamide/timolol, brimonidine, bimatoprost	Ahmed valve
11	PEX G	81	Male	Left	33	Dorzolamide/timolol, brimonidine, bimatoprost	Ahmed valve
12	PEX G	70	Male	Left	36	Dorzolamide/timolol, brimonidine, bimatoprost	Trabeculectomy

*IOP* intraocular pressure, *NTG* normal tension glaucoma, *PEX* pseudoexfoliation, *G* glaucoma, *Phaco* + *PCL* phacoemulsification and posterior intraocularlens insertion, *Ahmed valve* Ahmed valve implantation.

### The representative image of extracellular vesicles from aqueous humor

**Figure 1:**
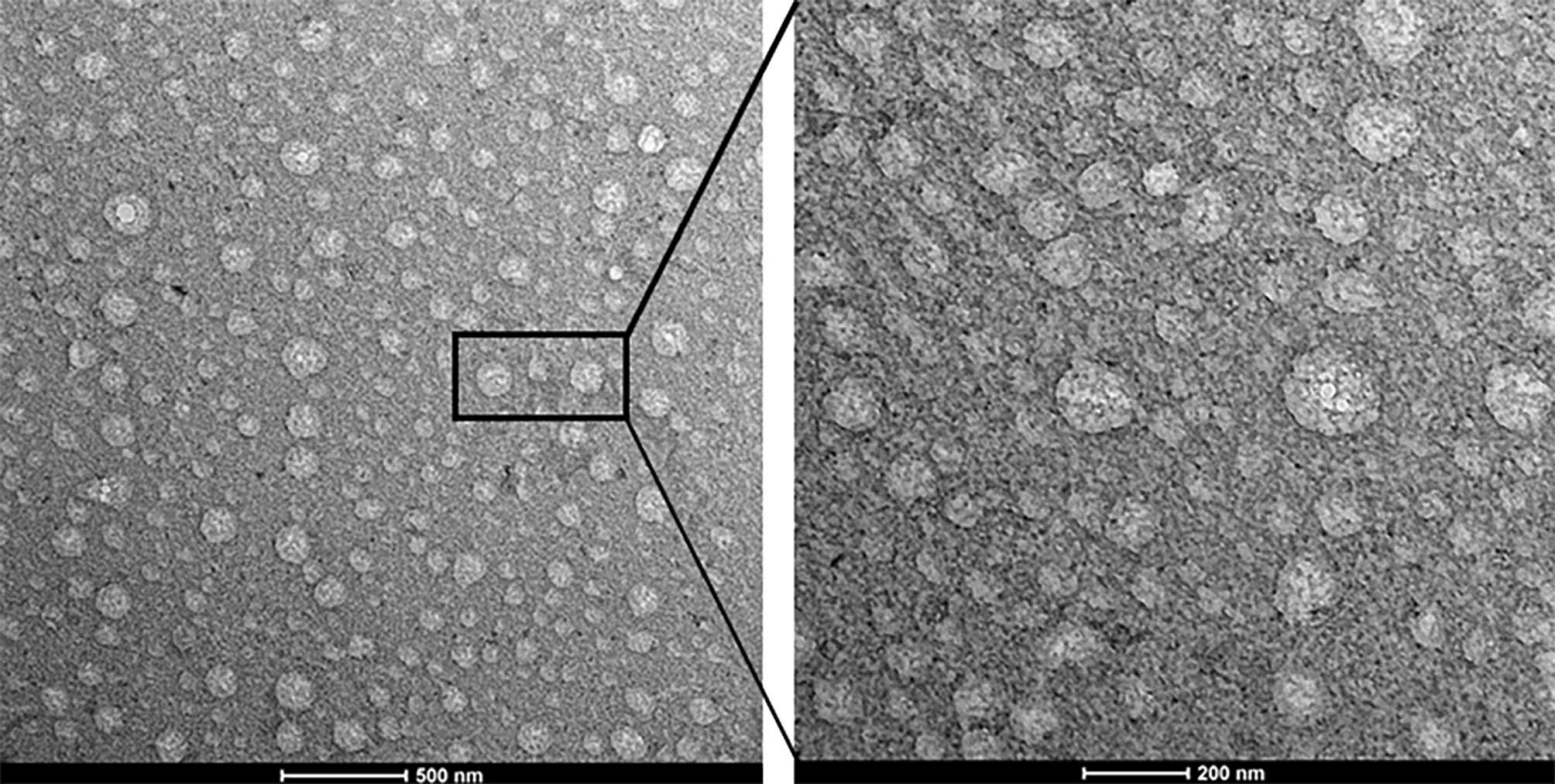
Transmission electron microscopic image of extracellular vesicles of aqueous humor. Numerous extracellular vesicles (EVs) with round shape morphology with various size (left, bar: 500 nm). On higher magnification, EVs up to 170 nm in size are present (right, bar: 200 nm).

The AH sample from a control subject was collected and the images of extracellular vesicles (EVs) were taken and visualized by transmission electron microscopy (TEM). EVs showed round shape morphology with size up to 170 nm. (Fig. 1) To gain further insight to analyze and characterize exosomes, we performed Exoview analysis to evaluate the size and particle counts of AH exosomes.

### Size distribution of the exosomes characterized by Exoview

The range and mean size distribution value of the exosomes in each CD63, CD81, CD9, and MIgG spot in the AH of the patients and corresponding control subjects ranged from 50 to 200 nm (mean = 54.92, 60.92, 57.58, and 62.17 nm, respectively). The mean size of each CD63, CD81, CD9, and MIgG spot in the control subjects was 54.67 ± 10.17 nm for CD63, 61 ± 22.17 nm for CD81, 56 ± 14.67 nm for CD9, and 65.33 ± 27.83 nm for MIgG. The mean size of each spot in the PEX patients was 55.17 ± 11.5 nm for CD63 (*p* = 0.319), 61.83 ± 24.17 nm for CD81 (*p* = 0.930), 56.17 ± 15.17 nm for CD9 (*p* = 0.200), and 65.67 ± 29.33 nm for MIgG (*p* = 0.250). There were no significant differences in the size distribution of the exosomes between the control group and the PEX glaucoma group (all *p* > 0.05).

**Figure 2:**
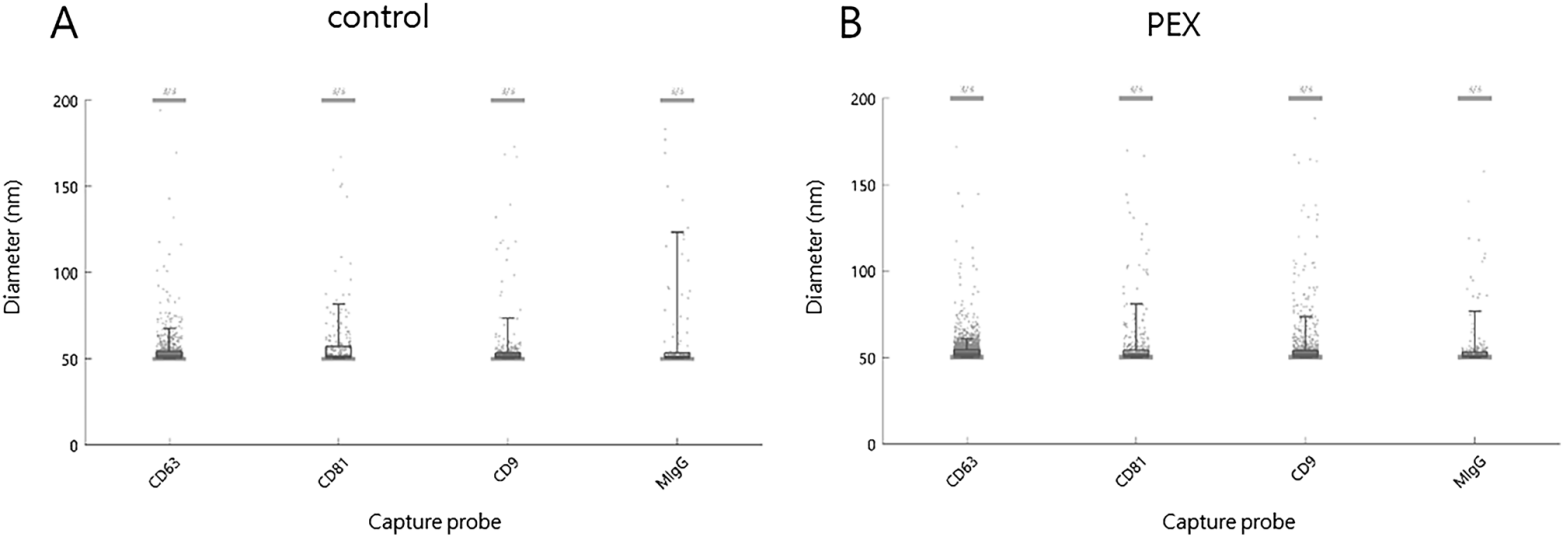
Quantitative exosome size distribution (**A**) Representative size distribution of AH control exosomes captured by the tetraspanin capture probe, including CD63, CD81, CD9, and MIgG is shown (55 ± 10 nm for CD63, 58 ± 17 nm for CD81, 55 ± 15 nm for CD9, and 62 ± 27 nm for MIgG). (**B**) Representative size distribution of AH exosomes from PEX patients captured (54 ± 60 nm for CD63, 56 ± 15 nm for CD81, 55 ± 13 nm for CD9, and 55 ± 13 nm for MIgG). Data are the mean ± standard deviation.

The representative size distribution of AH exosomes from control subject captured by the tetraspanin capture probe, including CD63, CD81, CD9, and MIgG, is shown in Fig. 2A. The mean ± standard deviation was 55 nm ± 10 nm for CD63, 58 ± 17 nm for CD81, 55 ± 15 nm for CD9, and 62 ± 27 nm for MIgG. The representative size distribution of AH exosomes from PEX patients is shown in Fig. 2B. The mean ± standard deviation was 54 ± 6 0 nm for CD63, 56 ± 15 nm for CD81, 55 ± 13 nm for CD9, and 55 ± 13 nm for MIgG.

### Exosome particle counts characterized by Exoview

**Figure 3:**
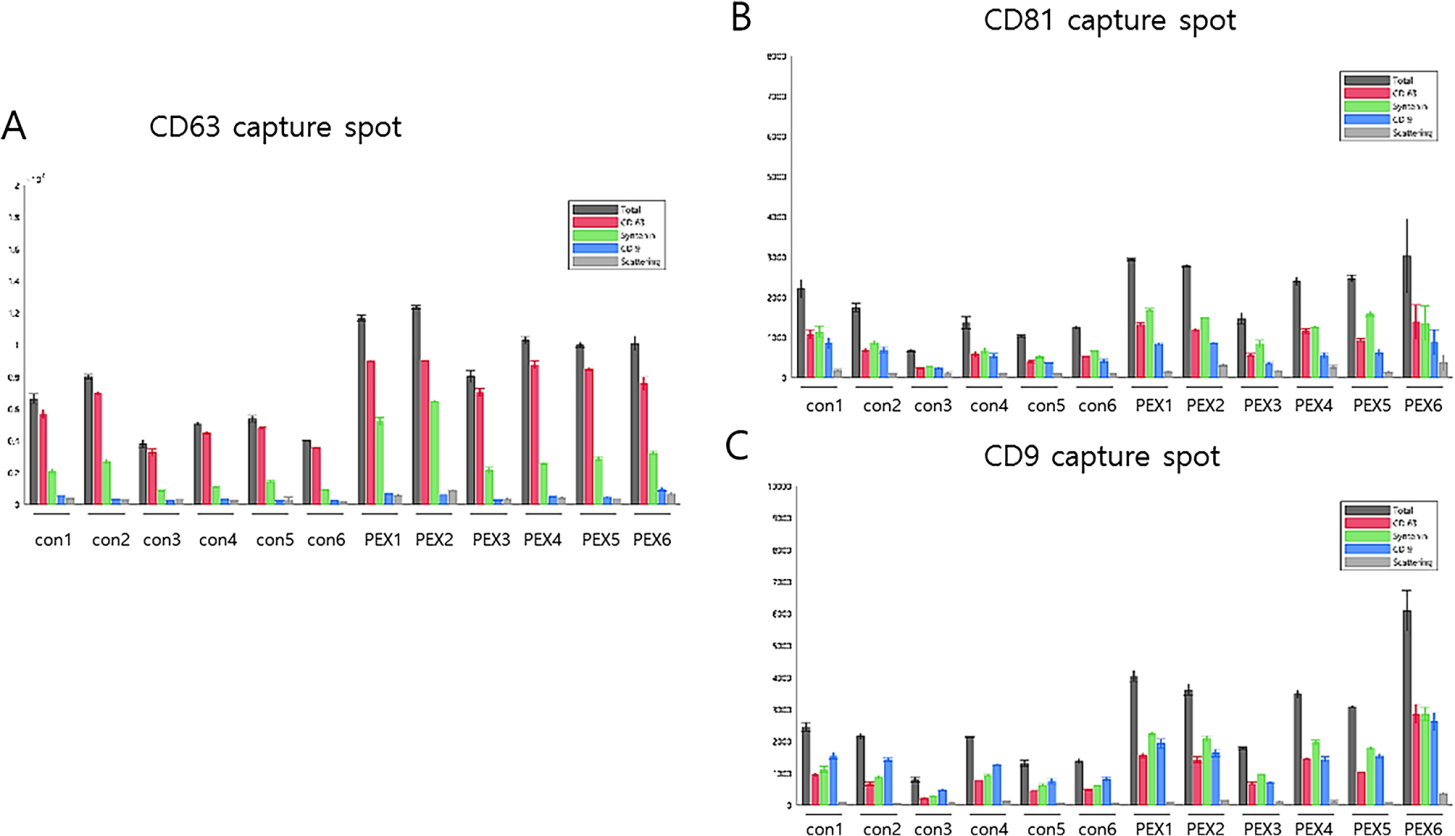
Quantitative exosome particle counts in individual patients The number of exosome particles detected by capture spots (**A**) CD63, (**B**) CD81, and (**C**) CD9 in one to six individual AH samples collected from PEX patients tended to be higher than in one to six control subjects.

**Figure 4:**
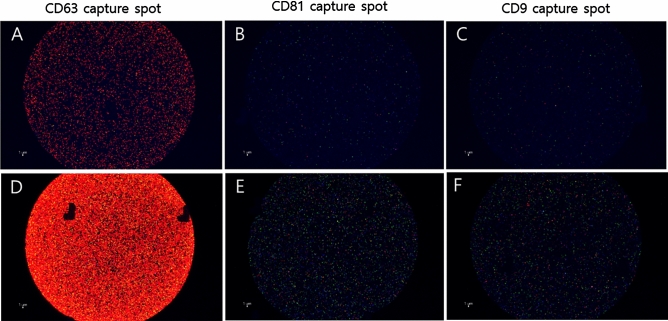
Representative capture spot images including CD63, CD81, and CD9 in (**A**–**C**) control subjects and (**D**–**F**) PEX patients are visualized. Compared to the control subjects (**A**–**C**), the number of the exosome particles in PEX patient AH samples (**D**–**F**) was higher. In addition, among each tetraspanin capture antibody detected in each capture spot, CD63 (red color) was present in the highest numbers of the exosomes, followed by syntenin (green color) and CD9 (blue color). In particular, syntenin with a green-colored capture antibody was detected in all three capture spots.

The exosome particle counts in individual patients and the corresponding control subjects were detected and counted according to each tetraspanin capture spot including CD63 (Fig. 3A), CD81 (Fig. 3B), and CD9 (Fig. 3C) and to each capture antibody including CD63, syntenin, and CD9. Compared to the controls, the numbers of exosome particles in the individual AH samples collected from PEX patients tended to be higher (Fig. 3A–C). Images of representative captured spots, including CD63, CD81, and CD9 for control subjects (Fig. 4A–C) and PEX patients (Fig. 4D–F) were visualized. Among each capture antibody detected on each tetraspanin capture spot, CD63 (red color) had the highest number of exosomes, followed by syntenin (green color) and CD9 (blue color). In particular, syntenin with a green-colored capture antibody was detected in all three capture spots. The average counts for EVs with each antibodies (CD63, syntenin, CD9, scattering, CD63/syntenin, CD63/CD9, syntenin/CD9, CD63/scattering, syntenin/scattering, CD9/scattering, CD63/syntenin/CD9, CD63/syntenin/scattering, CD63/CD9/scattering, CD63/syntenin/CD9/scattering) with colocalization of dual, triple, or quadruple-labelled EVs on each tetraspanin chip (CD63, CD81, CD9 and MIgG) were evaluated. The average colocalization counts for EVs in the AH of (A) representative control and (B) PEX glaucoma patient are visualized as the heatmap in the Supplementary Information.

In the grouped clinical data, the average total number of exosome particles in AH PEX samples was 10,414.83 ± 1426.774 for CD63, 3685.278 ± 1347.249 for CD81, and 2514.833 ± 625.0084 for CD9, which was higher and significantly different than the control subjects (5480.389 ± 1522.267 for CD63, 1707.556 ± 604.3676 for CD81, 1378.056 ± 518.0649 for CD9); *p* = 1.06892E−11 for CD63, *p* = 2.22692E−06 for CD81, and *p* = 7.56536E−06 for CD9).

**Figure 5:**
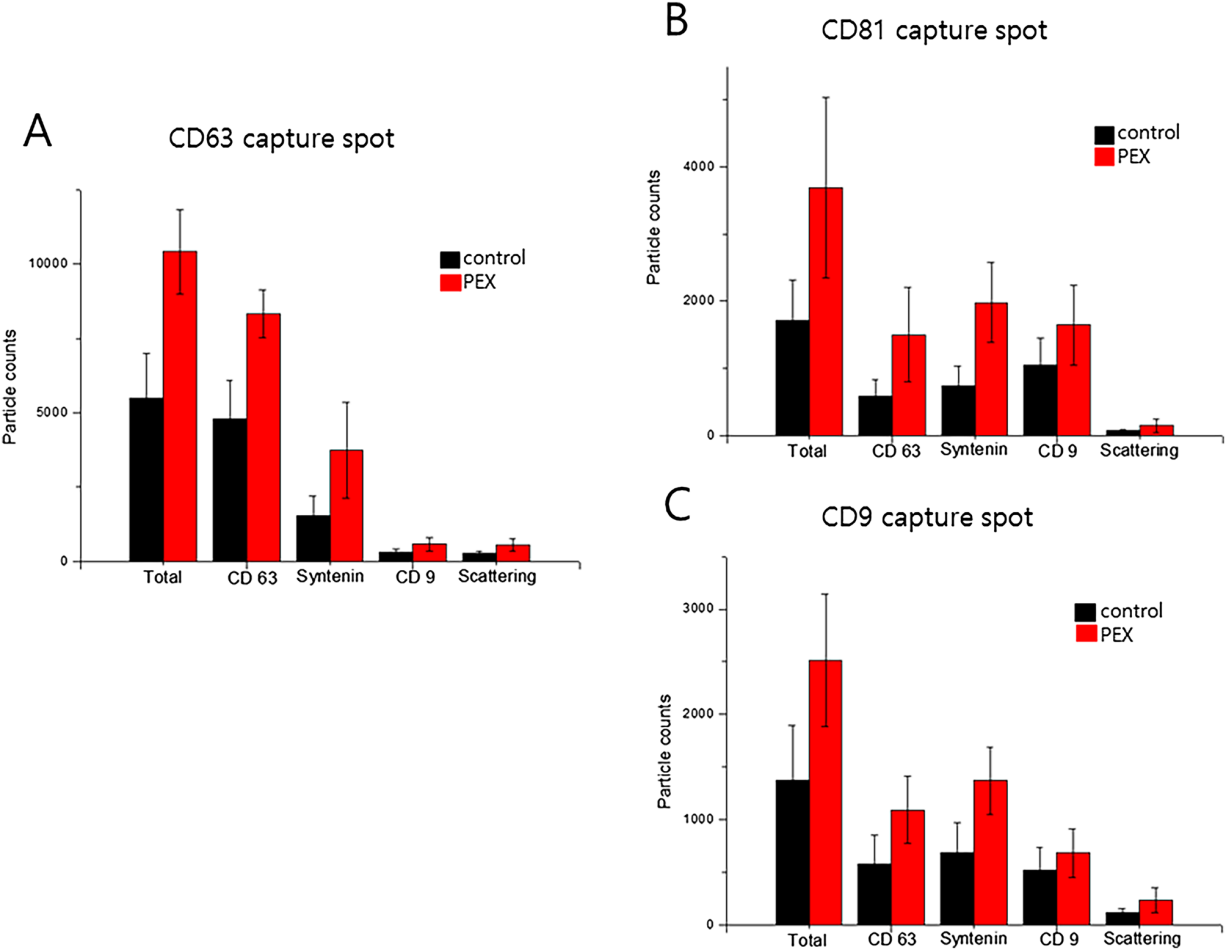
The number of exosome particles in grouped clinical data. Compared to the control subjects (black-colored in the bar graph), the numbers of the exosome particles in PEX patient AH samples (red-colored in the bar graph) were higher, with statistically significant differences. Capture spot CD63 (**A**), capture spot C81 (**B**), and capture spot CD9 (**C**).

The particle counts of each capture antibody are shown in Fig. 5 and Table 2. For capture spot CD63 (Fig. 5A), the *p*-value for CD63 was 1.88E−11, and it was 6.03E−06 for syntenin and 0.000115 for CD9. For capture spot CD81 (Fig. 5B), the *p*-value for CD63 was 1.05546E−05, and it was 2.60321E−09 for syntenin and 0.001288806 for CD9. For capture spot CD9 (Fig. 5C), the *p*-value for CD63 was 4.65406E−05, and it was 1.67314E−06 for syntenin and 0.031020409 for CD9 (Table 2).

**Table 2:** Exosome particle count per each spot between control and PEX glaucoma patients.

Capture spot	Capture antibody	Control (*n* = 6)	PEX G (*n* = 6)	*p*-value
CD63	Total	5480.389 ± 1522.267	10,414.83 ± 1426.774	**1.07E−11**
	CD 63	4786.778 ± 1302.085	8319.111 ± 797.6906	**1.88E−11**
	Syntenin	1512.556 ± 692.9516	3734.778 ± 1620.373	**6.03E−06**
	CD 9	312.5 ± 109.7791	569.1667 ± 224.5577	**0.000115**
	Scattering	266.1667 ± 83.86632	543 ± 203.5725	**6.32E−06**
CD9	Total	1378.056 ± 518.0649	2514.833 ± 625.0084	**7.56536E−06**
	CD 63	579.7222 ± 275.9712	1091.333 ± 321.8783	**4.65406E−05**
	Syntenin	689.9444 ± 282.7952	1372.444 ± 320.5819	**1.67314E−06**
	CD 9	516.1111 ± 220.7308	683.8889 ± 229.8593	**0.031020409**
	Scattering	116.3333 ± 35.26371	233.4444 ± 115.1742	**6.48256E−05**
CD81	Total	1707.556 ± 604.3676	3685.278 ± 1347.249	**2.22692E−06**
	CD 63	590.1667 ± 245.9087	1501.167 ± 707.0574	**1.05546E−05**
	Syntenin	744.8889 ± 279.5393	1979.722 ± 593.0894	**2.60321E−09**
	CD 9	1047.611 ± 402.4272	1645.111 ± 599.9681	**0.001288806**
	Scattering	71.11111 ± 24.07559	147.2778 ± 101.2439	**0.003820417**

Bold font indicates significant *p* values (*p* < 0.05). *p*-value by t-test.

*PEX* pseudoexfoliation, *G* glaucoma.

## Discussion

To the best of our knowledge, the present study was the first to report significantly differentially detected exosomes between controls and a specific type of glaucoma (PEX glaucoma) in individual AH samples without pooling, using a novel detection platform based on a single-particle interferometric reflectance imaging sensor and a tangential flow filtration system-based method. The current study has significant meaning in that the quantitative exosome particle count in each fluorescent capture spot image was analyzed in each AH sample. The PEX glaucoma AH samples had significantly higher exosome particle counts than those in the control group. The fluorescent capture spot images also revealed denser exosome particles in the individual images in PEX patients than in control subjects. The particle size measurement was also quantitatively provided with actual mean and standard deviation values. Moreover, the current study is unique in that all included subjects were Koreans and it was conducted in a single ethnic group. The present study not only detected exosomes in AH but also investigated exosome cargoes including syntenin. The results confirmed that the particles detected in AH were genuine exosomes. These findings suggest the functional participation of exosomes in the pathophysiology of glaucoma, particularly PEX glaucoma.

The term “exosome” was first used in the early 1980s to describe small (30 – 100 nm) vesicles of endosomal origin secreted by reticulocytes^22,23^. According to Kowal et al., small vesicles (diameter ≈ 30–150 nm) with a low density in gradient ultracentrifugation and carrying tetraspanins (CD63, CD81, and CD9), syntenin-1, and TSG101 proteins, qualified as exosomes^24^. The role of exosomes in eye and visual system disorders has been described in several reports including review articles^25,26^. Exosomes are consisted of numerous molecules capable of cellular signaling, which may be used for therapy or for analysis of biomarker. Exosomes are released from cells through the whole body and contain lipids, proteins, carbohydrates, cytokines, miRNA, and RNA cargo. Exosomal surface proteins, carbohydrates, and lipids direct cellular targeting, and can initiate cell surface signaling and affect the mechanism and location of internalization^26^. Therefore, exosomal molecular profiles and cargo are attractive biomarkers for optic nerve diseases such as glaucoma^26^.

Several methods including flow cytometry, nanoparticle tracking analysis, and transmission electron microscopic analysis have been used to detect and characterize exosomes. Although conventional flow cytometry of bead-captured vesicles has been used as a convenient approach for multi-parametric and high-throughput characterization of individual cells, it only detects sizes greater than 500 nm. Since small EVs including exosomes were reported to range from 50 to 250 nm in size, advanced imaging flow cytometry combined with exosome-specific monoclonal antibodies has been suggested to supplement conventional flow cytometry analysis^27^. Meanwhile, nanoparticle tracking analysis (NTA) is a method for analyzing and visualizing particles in liquids that associates the rate of Brownian motion to particle size. Currently, NTA is used for particles from about 10 to 1000 nm in size and has been used for characterizing exosomes^12,21^. However, there are some limitations. The viscosity of the solvent affects the movement of particles, thus playing a role in establishing the upper size limit, and exosomes should be isolated as pellets for characterization using NTA. Therefore, in this study, we used ExoView®, a novel detection platform based on a single-particle interferometric reflectance imaging sensor (SP-IRIS). Exoview can directly detect exosomes in samples including AH, serum, plasma, urine, or cultured cells without sample purification. Moreover, the volume of the sample required for analyzing exosome size and particle counts using Exoview is much smaller than that for NTA. Exoview uses 35 μL compared to 600 μL for NTA. The minimal sample volume might be the most interesting and distinguishing point since the amount of AH obtained during surgery can be very small. Among other options for Exoview analysis, in this study, we used the Exoview Tetraspanin Cargo kit to characterize human AH exosomes. Importantly, the reason we used the cargo protein syntenin was that it is often offered as proof of the multivesicular body (MVB) origin of exosomes. Syntenin PDZ domains directly bind to multiple syndecan membrane receptors and the tetraspanin CD63 to support exosomal cargo sorting^28^. Only after sorting, do genetic materials, including miRNAs and other cargo proteins, enter the MVB to differentiate into exosomes.

This exploratory study was limited by the relatively small volume of the AH samples and small sample size. Our results may not represent the whole Asian ethnicity but limited to Korean population. However, it has a significance in that it provides the potential for future research in the field of exosomes in eye disorders, especially, glaucoma. The effect of hypotensive topical medications on AH exosomal profiles and exosomal miRNA expression in PEX glaucoma patients has not been revealed yet. The influence of various hypotensive topical medications on our results is unknown. Future studies including large numbers of samples would be beneficial in controlling the use of topical medications. Nevertheless, glaucoma patients generally take hypotensive anti-glaucoma topical medications, particularly when they decide to undertake ocular surgery except they are discovered to be medication-naïve in the clinic. Because it is not ethical to acquire the AH of patients that are not undergoing ocular surgery in operating rooms or stop medications for glaucoma patients for research purposes, it is not simple to eliminate the effect of hypotensive anti-glaucoma topical medications on the results. In the same context, PEX glaucoma patients with high IOP using multiple hypotensive medications who needed filtering surgery were included in the present study. The effect of including PEX patients with high IOP on the study results is unknown. High IOP and/or the use of hypotensive medications may affect the blood-aqueous-barrier, which may further affect the composition of exosomes. However, according to a recent review article, compromised aqueous-blood barrier was also one of mechanisms involved in the pathogenesis of PEX syndrome and PEX glaucoma^29^. PEX syndrome usually does not show high IOP or glaucomatous damage in the optic nerve head. Therefore, hypotensive medication is not needed in most cases. However, even eyes with PEX syndrome frequently show clinical signs of impairment of the blood-aqueous-barrier^30^. The breakdown of the blood-aqueous-barrier is confirmed by an elevation in AH proteins^30^. Therefore, the disruption of aqueous-blood barrier is not thought to be only affected by high IOP or hypotensive medications, but PEX glaucoma itself also has the property of compromised aqueous-blood barrier. We think the significant difference of exosomes between PEX glaucoma and control shown in the current study is likely due to the own disease of PEX glaucoma.

In conclusion, exosomes from individual AH samples showed a significant difference between PEX glaucoma and control groups in a single ethnic group of Koreans. Exosomes in AH quantitatively assessed by the novel detection platform method revealed significantly higher numbers of exosomes in the PEX glaucoma group compared to the controls, which has not been reported before. Our findings suggest the possible role of exosomes and exosomal cargos in the pathogenesis of PEX glaucoma. Our results also further imply the potential of exosomes as a biomarker for glaucoma, especially PEX glaucoma for diagnosing and providing patient prognoses. Further studies with more numbers of samples are required to draw definitive conclusions.

## Methods

### Ethics statement

The present study was performed according to the tenets of the Declaration of Helsinki for research concerning human subjects. The present study was approved by the Institutional Review Board of Gyeongsang National University Changwon Hospital, Gyeongsang National University, School of Medicine (GNUCH-2019-06-001-002). Written informed consent was obtained from all subjects included in the present study. All methods were carried out in accordance with relevant guidelines and regulations.

### Diagnosis of PEX glaucoma

Subjects were assessed in the glaucoma clinic at Gyeongsang National University Changwon Hospital by a single glaucoma specialist (H.-K. C.). PEX glaucoma was defined by the existence of PEX material at the margin of the pupil and on the anterior lens capsule after maximal pupil dilatation, and all of the following: an initial IOP of at least 22 mmHg, glaucomatous optic disc changes, visual field defects corresponding to optic nerve damage, and no evidence of other conditions causing secondary glaucoma^7^. All subjects underwent standard ophthalmic examinations including slit-lamp biomicroscopy, gonioscopy, and funduscopy.

### Patient selection and acquisition of aqueous humor samples

AH samples were obtained from patients who underwent uneventful phacoemulsification for elective cataract surgery or glaucoma filtering surgery (trabeculectomy or Ahmed glaucoma drainage implant surgery) after obtaining written informed consent. Six PEX glaucoma patients and six age-matched control subjects agreed to take part in the present study. About 80 to 120 μL of AH was obtained by anterior chamber paracentesis with a 30-gauge needle prior to the main corneal incision at the beginning of cataract surgery or during the procedure of paracentesis in trabeculectomy or Ahmed glaucoma drainage implant surgery. Anterior chamber paracentesis was performed under aseptic sterile conditions in the operating room. AH was acquired without trauma to the subjects, thus, excluding any possibility of contamination with blood or cellular debris. All obtained AH samples were anonymized, promptly snap-frozen with liquid nitrogen, and transferred to research laboratories. Clinical data were collected from electronic medical records in a completely anonymized method. The acquired clinical data were age, eye laterality, sex, baseline IOP, use of topical medications, and other ocular comorbidities.

### Transmission electron microscopic analysis

10 ul of AH collected form the representative control patient was fixed 1:1 with 2% glutaraldehyde (Tokyo Chemical Industry, G0068) for 30 min. The fixed sample of 6 μL was pipetted onto the 200 mesh copper grid with carbon-coated formvar film, and incubated for 10 min. The grid was washed with 100 μL MilliQ water. The grid was placed on 30 μL of 1.5% uranyl acetate for 12 s. Images were acquired using Bio 120 kV Transmission electron microscope (Thermofisher Scienfitic, Talos L120C)^31^.

### ExoView analysis

The Exoview platform consists of an antibody array to isolate exosomes on the chip based on surface membrane-bound markers and an interferometric imaging technique to detect exosomes using fluorescence. Using the multiplexed microarray, the particle diameter of single exosomes was measured by their light scattering intensity. In addition, exosomes expressing specific surface membrane-bound markers, including tetraspanins (CD63, CD81, and CD9) were counted using three-color fluorescence. Exosome cargo, luminal proteins including ALIX and syntenin, could be probed simultaneously with surface markers using a permeabilization assay. As these methods relied on affinity capture, which could directly detect exosomes in the samples and avoid certain complications and biases associated with sample processing^32,33^.

The biological and physical properties of exosomes in human AH samples were characterized using Exoview R-100 (Nanoview Bioscience, Boston, MA, USA), ExoView Tetraspanin kits (NanoView Biosciences) including anti-CD81, anti-CD63, anti-CD9, and IgG negative control-immobilized chips, fluorescence-labeling agents, washing solutions (solution A and B) and blocking solution (NanoView Biosciences), the ExoView Tetraspanin Cargo kit, including anti-syntenin antibody, and cargo staining blocking solution, solution C, and solution D. Briefly, 35 uL of sample diluted with solution A was dropped onto Exoview tetraspanin chips and incubated overnight (16 h) at room temperature. After incubation, the sample-loaded chip was washed with 1 mL of solution A, 250 mL of solution C, and 250 mL of solution D 3 times for 3 min. After the last wash, the exosomes on the chip were labeled with 250 uL of a fluorescently labeled antibody mixture of anti-syntenin/AF 555 (green), anti-CD 63/AF 647 (red), and anti-CD 9/AF 488 (blue) and incubated for 1 h at room temperature. The co-localization of tetraspanin on the surface and the exosomal cargo were analyzed. During the process, anti-CD 63/AF 647 and anti-CD 9/AF 488 were diluted at 1:500, and Syntenin/AF 555 was diluted at 1:200 in a mixture of solution A and cargo blocking solution. Finally, the chip was rinsed with 1 mL of solutions A and B 3 times for 3 min and dried at room temperature. The exosome capture chip was scanned using Exoview R-100 and nScan software and the data were analyzed by Exoview Analyzer 3.0 software.

### Statistical analysis

Comparison of exosomal particle counts between PEX glaucoma and control groups was performed with the unpaired Student’s t-test (Prism 5; GraphPad Software, La Jolla, CA, USA). A *p*-value of < 0.05 indicated a statistically significant difference.

## Supplementary Information

Supplementary Information.

## Data Availability

The datasets used in the current study might be shared upon reasonable request to Hyun-kyung Cho, MD, PhD.
