# Supplementary material for: Quantitative analysis of exosomes in the aqueous humor of Korean patients with pseudoexfoliation glaucoma
Source: Sci Rep. 2022 Jul 27;12:12875. doi: 10.1038/s41598-022-17063-9 (PMC9329372; doi:10.1038/s41598-022-17063-9)
Supplement: Supplementary file 1 — Supplementary Information. [file 41598_2022_17063_MOESM1_ESM.docx]

Supplementary information

**Quantitative Analysis of Exosomes in the Aqueous Humor of Korean Patients with Pseudoexfoliation Glaucoma**

Hyo Jung An MD, PhD,^1,2^ Hyun-kyung Cho, MD, PhD,^2,3^ Dae Hyun Song, MD, PhD,^1,2^ Changwon Kee, MD, PhD^4^

^1^Department of Pathology, Gyeongsang National University Changwon Hospital, Gyeongsang National University, School of Medicine, Changwon, Republic of Korea, ^2^lnstitute of Health Sciences, School of Medicine, Gyeongsang National University, Jinju, Republic of Korea, ^3^Department of Ophthalmology, Gyeongsang National University Changwon Hospital, Gyeongsang National University, School of Medicine, Changwon, Republic of Korea, ^4^Department of Ophthalmology, Samsung Medical Center, Sungkyunkwan University School of Medicine, Seoul, Republic of Korea

Short title: Exosomes in aqueous humor of pseudoexfoliation glaucoma patients

Correspondence: Hyun-kyung Cho, MD, Ph.D.

Department of Ophthalmology, Gyeongsang National University Changwon Hospital, Gyeongsang National University, School of Medicine, 11 Samjeongja-ro, Seongsan-gu, Changwon, Gyeongsangnam-do, 51472, Republic of Korea

Tel: +82-55-214-2410; Fax: +82-55-214-3257; E-mail: chohk@gnu.ac.kr/ kanojo99@hanmail.net

Supplementary information

Average colocalization counts for exosomes in the aqueous humor. (A) Colocalization count of exosomes from the aqueous humor of the representative control patient. Among each tetraspanin and syntenin antibodies, colocalization count of CD63 antibody on CD63 tetraspanin chip was the highest, 2515 out of 3804 total counts (red box). (B) Colocalization count of exosomes from the aqueous humor of the representative PEX glaucoma patient. Among each tetraspanin and syntenin antibodies, colocalization count of CD63 antibody on CD63 tetraspanin chip was the highest, 5083 out of 12356 total counts (red box), and CD63/syntenin (3359), and syntenin (2482) in numeric order.


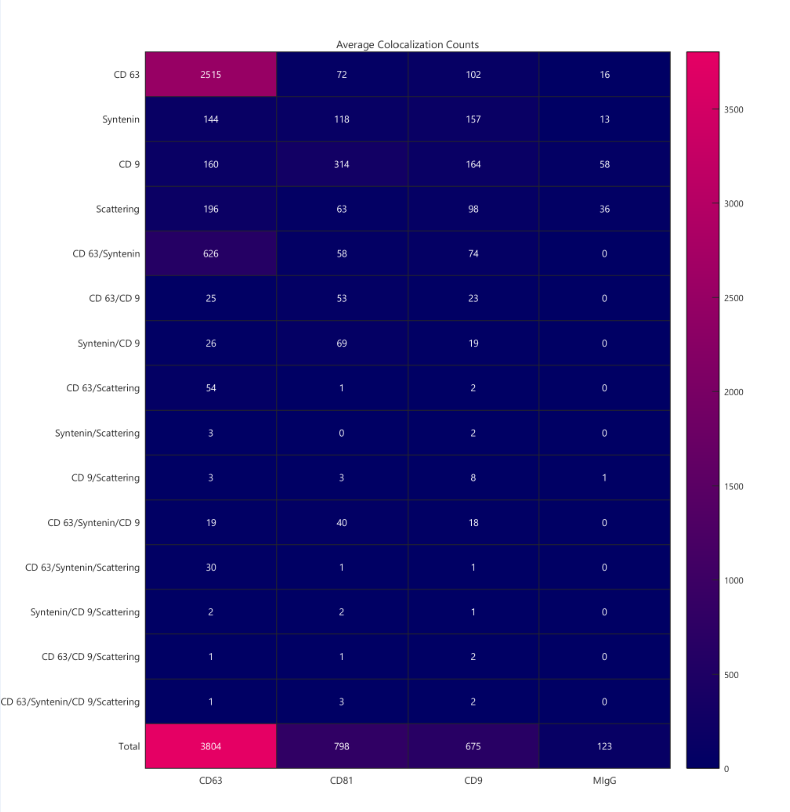


A


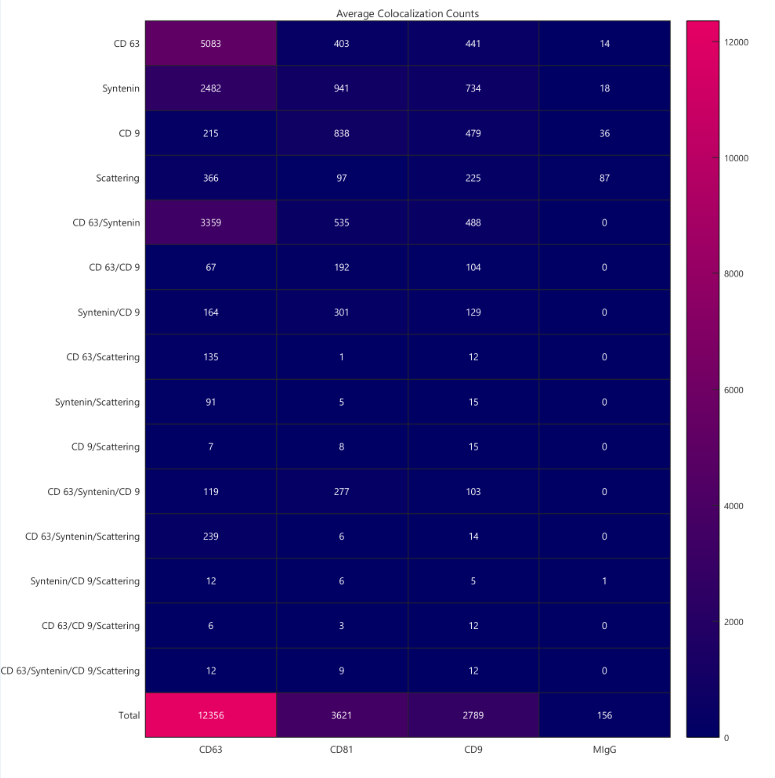


B
